# Identification and Network Construction of mRNAs, miRNAs, lncRNAs, and circRNAs in Sweetpotato (*Ipomoea batatas* L.) Adventitious Roots Under Salt Stress via Whole-Transcriptome RNA Sequencing

**DOI:** 10.3390/ijms26041660

**Published:** 2025-02-15

**Authors:** Bo Jiang, Yuxia Li, Jun Shi, Dagaga Dibaba Chalasa, Lei Zhang, Shaoyuan Wu, Tao Xu

**Affiliations:** Jiangsu Key Laboratory of Comparative Genomics, School of Life Sciences, Jiangsu Normal University, Xuzhou 221116, China

**Keywords:** whole-transcriptome RNA sequencing, lncRNAs, miRNAs, circRNAs, salt stress, sweetpotato

## Abstract

Sweetpotato is the seventh largest crop worldwide, and soil salinization is a major environmental stress limiting its yield. Recent studies have shown that noncoding RNAs (ncRNAs) play important regulatory roles in plant responses to abiotic stress. However, ncRNAs in sweetpotato remain largely unexplored. This study analyzed the characteristics of salt-responsive ncRNAs in sweetpotato adventitious roots under salt stress via whole-transcriptome RNA sequencing. The results revealed that 3175 messenger RNAs (mRNAs), 458 microRNAs (miRNAs), 544 long-chain ncRNAs (lncRNAs), and 23 circular RNAs (circRNAs) were differentially expressed. Kyoto Encyclopedia of Genes and Genomes (KEGG) enrichment analysis revealed that most differentially expressed mRNAs (DEmRNAs) and miRNAs (DEmiRNAs) were enriched primarily in phenylpropanoid biosynthesis, starch and sucrose metabolism, the Mitogen-Activated Protein Kinase (MAPK) signaling pathway, plant hormone signal transduction, the mRNA surveillance pathway, and ATP-binding cassette (ABC) transporters. Gene Ontology (GO) enrichment analysis revealed that the majority of DEmRNAs, their target DEmiRNAs, and differentially expressed lncRNAs (DElncRNAs) were associated with the cell wall, oxidation–reduction, the plasma membrane, protein phosphorylation, metabolic processes, transcription factor activity, and the regulation of transcription. Additionally, based on the competitive endogenous RNA (ceRNA) hypothesis, we predicted interactions among different RNAs and constructed a salt-responsive ceRNA network comprising 22 DEmiRNAs, 42 DEmRNAs, 27 DElncRNAs, and 10 differentially expressed circRNAs (DEcircRNAs). Some miRNAs, such as miR408, miR169, miR160, miR5139, miR5368, and miR6179, were central to the network, suggesting their crucial roles in the sweetpotato salt response. Our findings provide a foundation for further research into the potential functions of ncRNAs and offer new targets for salt stress resistance improvement through the manipulation of ncRNAs.

## 1. Introduction

Soil salinization, a significant environmental stress, affects approximately 831 million hectares of land across more than 100 countries [1,2]. Projections indicate that by 2050, salinization will impact half of the world’s agriculturally cultivated land [3]. High soil salinity can lead to a range of issues for plants, including ion imbalance, compromised infiltration, oxidative stress, and secondary challenges, ultimately restricting crop growth and agricultural yield [4]. In response to salinity stress, plants have developed various mechanisms, including the use of aquaporins [5], transport proteins [6], and a diverse array of enzymes that play critical roles in neutralizing free radicals. Research has identified specific genes that are vital to the plant response to salt stress, as they engage in multiple signaling pathways, including those regulated by Ca^2+^ [7], abscisic acid (ABA) [8,9], ethylene [10], phospholipids [11], and the MAPK cascade [12]. ABA-responsive element-binding factors (ABFs/AREBs) are involved in regulating the ABA signaling pathway under salt stress conditions [13]. In the final stage of ethylene synthesis, ACC oxidase (ACO) converts ACC into ethylene, contributing to the response of plants to salinity [14]. Calcium-dependent protein kinases (CDPKs), which are proteins that bind Ca^2+^, play a key role in signaling pathways associated with both salt stress and ABA [15,16]. Furthermore, in addition to protein-coding RNAs, noncoding RNAs (ncRNAs), such as miRNAs, circRNAs, and lncRNAs, are involved in gene regulation and are essential for plants to respond effectively to stress conditions [17,18,19].

MiRNAs, which are endogenous ncRNAs ranging from 18 to 25 nucleotides (nt) in length, play crucial regulatory roles in eukaryotes [20,21,22]. They recognize target mRNAs through complementary base pairing, thereby influencing post-transcriptional processes by either degrading these mRNAs or inhibiting their translation [23,24,25]. A growing body of research indicates that miRNAs are integral to the response to salt stress across various plant species [26,27,28,29,30,31]. In crop studies, the overexpression of *osa-miR171c* or *osa-MIR396c* in rice has been shown to reduce salt stress tolerance [32,33]. Conversely, miR2871b negatively influences salt stress tolerance in transgenic rice plants [34], whereas the suppression of miR168 enhances salt tolerance in rice [35]. In maize, miR408 has been associated with reduced salt tolerance due to its effect on secondary cell wall formation [36], whereas miR169q promotes the expression of *PEROXIDASE1*, thereby improving salt tolerance [37]. In soybean, the miR172c-NNC1 module is critical for adjusting root plasticity under saline conditions [38], and the overexpression of miR172c has been shown to increase salt stress tolerance [39]. Additionally, in cotton, miR414c affects salt tolerance by modulating reactive oxygen metabolism [40]. Moreover, miR408 influences the signaling pathway and osmoprotective biosynthesis in wheat under saline conditions [41], and the overexpression of *miR156* confers salt stress resistance in alfalfa [42].

LncRNAs, which are ncRNAs exceeding 200 nts in length, also play significant roles in plant stress responses [43,44]. They are involved in various processes, including epigenetic modification, as well as transcriptional and post-transcriptional regulation [45]. Through whole-transcriptome RNA sequencing, researchers have identified 505, 44, and 185 lncRNAs in response to salinity conditions in *Medicago truncatula* [46], *Gossypium hirsutum* [47], and *Spirodela polyrhiza* [48], respectively. In cotton, lncRNA973 regulates numerous genes, including those associated with reactive oxygen-scavenging [49]. Additionally, a study on a hyperarid maize variety exposed to simultaneous salt and boron stress identified a total of 1710 putative lncRNAs [50]. Lv et al. identified 1077 DElncRNAs and reported that 39 lncRNAs serve as key hubs in the plant response to abiotic stress [51]. These findings collectively suggest that lncRNAs are actively involved in plant adaptation to salt stress.

CircRNAs are a unique class of endogenous ncRNAs characterized by their covalently closed circular structure [52]. These circRNAs possess numerous binding sites for miRNAs, enabling them to sequester cytoplasmic miRNAs and mitigate the repressive effects of miRNAs on their target mRNAs [53,54,55,56,57]. Recent studies have indicated that circRNAs may play a significant role in enhancing the ability of plants to withstand stress. For example, chilling stress affects 163 circRNAs in *Solanum lycopersicum* [58]. Additionally, 33 and 62 DEcircRNAs were identified in birch-leaf pear and the leaves of wheat seedlings, respectively, when subjected to dehydration stress [59,60]. In *Populus euphratica*, 18 circRNAs are associated with the stress response [61]. Furthermore, in *Arabidopsis*, the overexpression of *Vv-circATS1* has been shown to increase plant cold tolerance [62]. Collectively, these findings suggest that circRNAs play a critical role in the adaptive response to abiotic stress.

As competing endogenous RNAs (ceRNAs), both lncRNAs and circRNAs bind competitively to miRNAs, resulting in changes to the target genes regulated by these miRNAs and facilitating post-transcriptional gene regulation [63,64,65]. Recent advancements in sequencing technologies and bioinformatics methods have led to the widespread implementation of ceRNA analysis as a novel framework for examining gene regulation through miRNA–lncRNA, miRNA–circRNA, and miRNA–mRNA interactions during plant responses to abiotic stress [66,67,68]. For example, ceRNA interactions have been explored in poplar for low nitrogen adaptation [69], in *Citrus junos* related to copper toxicity [70], in tomato and *M. truncatula* under salt stress [14,71], and in cucumber during heat stress [68]. However, a comprehensive analysis of mRNAs, miRNAs, lncRNAs, circRNAs, and their ceRNA networks in sweetpotato is lacking.

Sweetpotato (*Ipomoea batatas* L.) is a vital global food crop recognized for its high yield and nutritional richness [72,73,74]. The role of miRNAs has been explored in sweetpotato recently. For example, miR828 has been shown to regulate lignin and hydrogen peroxide (H_2_O_2_) accumulation [75], whereas miR408 is involved in the defensive response to wounding in sweetpotato [76]. Additionally, the downregulation of miR2111 results in an increase in *IbFBK* (*Fbox/kelch repeat protein*), which may affect the protein degradation of *IbCNR8* (*cell number regulator 8*) during wounding responses in sweetpotato [77]. Furthermore, a total of 121 DEmiRNAs were identified between white-fleshed and purple-fleshed sweetpotato varieties [78]. In the context of salt stress, 13 miRNAs were significantly upregulated, whereas 9 miRNAs were substantially downregulated in sweetpotato roots subjected to salt stress [79]. A comparative study of miRNAs responsive to salt stress was conducted on two sweetpotato cultivars that display differing levels of salt stress resistance [80]. However, the roles of ncRNAs, including miRNAs, circRNAs, and lncRNAs, in sweetpotato still remain largely unknown.

Recently, a lot of evidence suggests that two types of ncRNAs, lncRNAs, and circRNAs, play important regulatory roles as ceRNAs in plant responses to abiotic stress. To date, there have been no scientific reports on stress-responsive circRNAs and lncRNAs in sweetpotato. In this study, we identified mRNAs, miRNAs, circRNAs, and lncRNAs that respond to salt stress in the adventitious roots of sweetpotato and constructed a salt-responsive ceRNA regulatory network. This work provides a theoretical foundation for future functional studies of ncRNAs under salt stress conditions in sweetpotato.

## 2. Results

### 2.1. Physiological Changes in Sweetpotato Roots Under Salt Stress

**Figure 1:**
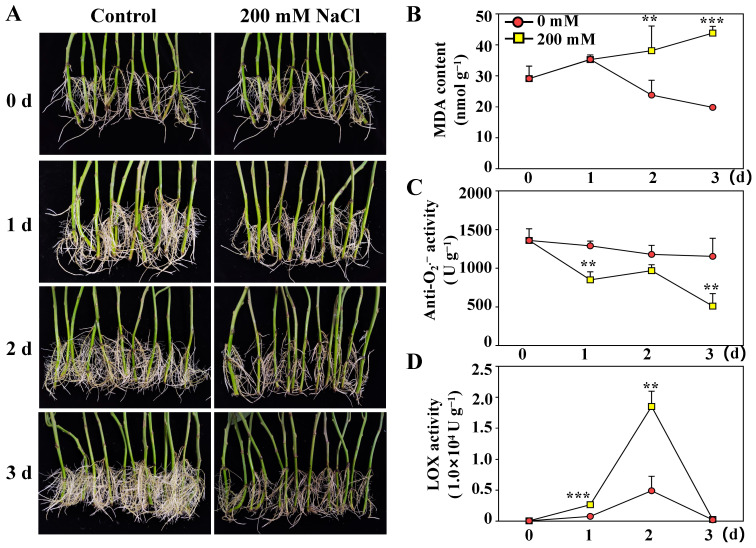
Salt stress (200 mM NaCl) inhibited the growth of sweetpotato roots and increased the accumulation of active oxygen in sweetpotato roots. (**A**) Phenotype of sweetpotato roots treated with 200 mM NaCl at 28 °C for three days. (**B**) MDA content. (**C**) Anti-O_2_^.−^ activity. (**D**) LOX activity. The values are expressed as the means of three replicates. The vertical bars represent the standard errors of the values of triplicate assays. All experiments were performed in three independent biological replicates on a fresh weight basis. Asterisks indicate that the values between the control and NaCl-treated roots were significantly different (** *p* < 0.01, *** *p* < 0.001).

As shown in Figure 1A, root growth was significantly inhibited after three days of NaCl treatment. On the first day, the sweetpotato roots slowly grew, and the root color became pale in the NaCl treatment group. By the second day, some new roots had formed in the control group. In contrast, the NaCl treatment group presented marked damage to both the proximal and distal sections of the roots, with colors shifting to yellow or even black, indicating severe inhibition of root growth due to NaCl exposure. After three days of NaCl treatment, the roots had taken on a blackened hue, and the root system in the treatment group demonstrated significantly less vigorous growth than that in the control group.

An increase in soil salinity leads to alterations in malondialdehyde (MDA) levels and enzyme catalytic activities in plants. To assess the response of sweetpotato roots to salt stress, we measured the levels of MDA, the activities of anti-superoxide radical (anti-O_2_^.−^), and lipoxygenase (LOX, EC.1.13.11.12). As depicted in Figure 1B, the MDA content in roots treated with NaCl was initially similar to that in control roots, but it increased consistently from the second day onward. The activity of anti-O_2_^.−^ decreased in both the control and NaCl-treated roots, with a more rapid decline observed in the salt-treated group (Figure 1C). As shown in Figure 1D, LOX activity in sweetpotato roots displayed a similar pattern in both the control and NaCl-treated groups. However, the LOX activity in NaCl-treated roots increased significantly during the first two days and subsequently decreased more quickly than that in the control roots.

### 2.2. Global Response of mRNAs to Salt Stress

**Figure 2:**
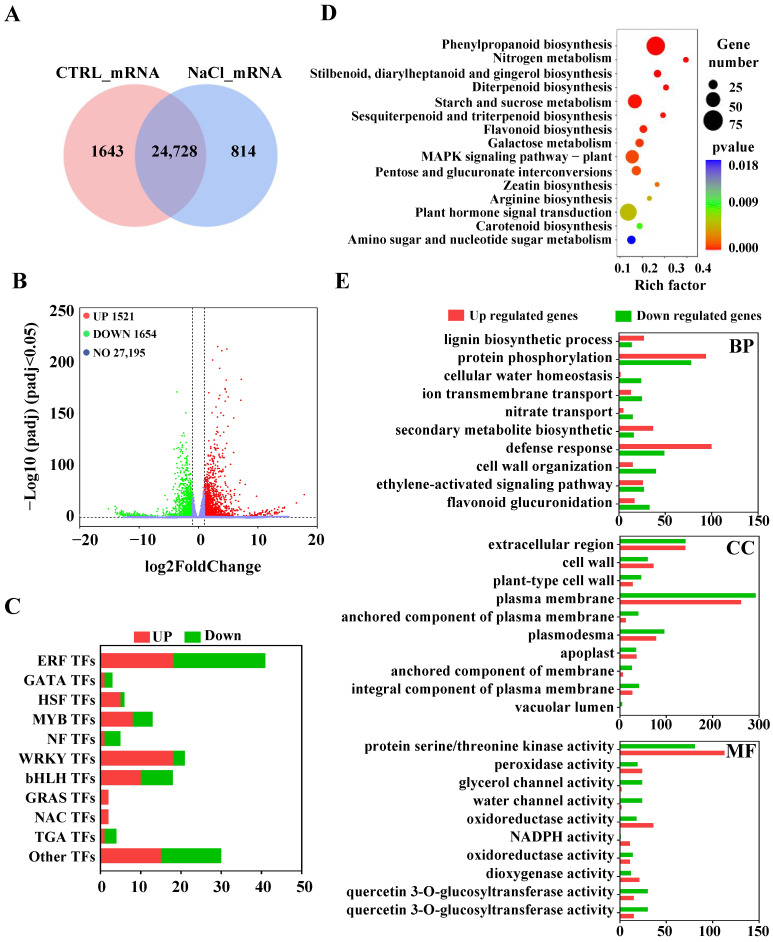
Identification and characterization of salt-responsive mRNAs in sweetpotato roots. (**A**) Venn diagram showing the distribution of the mRNAs in CTRL/NaCl. (**B**) Volcano plot of detected mRNAs. Red and green represent the upregulated and downregulated mRNAs under salt stress, respectively. (**C**) The top 15 differentially expressed transcription factor families in sweetpotato under salt stress. (**D**) Top 20 enriched KEGG pathways of DEmRNAs in sweetpotato roots. (**E**) GO classification of DEmRNAs in sweetpotato roots.

A total of six libraries, including those from the control and NaCl-treated groups, were constructed from sweetpotato roots, generating 491,265,684 raw reads through RNA sequencing (Table S1). After low-quality and linker sequences were filtered out, 439,823,566 clean reads were retained for further analysis. These reads were mapped to the sweetpotato (*Ipomoea trifida*) genome, with an average mapping ratio exceeding 65.47%. The expression levels of genes were quantified as fragments per kilobase of exon per million mapped reads (FPKM), identifying 31,891 mRNAs as expressed genes (Table S2). The Venn diagram revealed that 1643 mRNAs were uniquely expressed in the control group, whereas 814 mRNAs were specifically expressed in NaCl-treated roots (Figure 2A). The volcano plot revealed 3175 DEmRNAs in sweetpotato roots (Figure 2B). Among these genes, 1521 were upregulated, and 1654 were downregulated in response to salt stress (Figure 2B, Table S3).

Additionally, we identified several transcription factor (TF) families (Figure 2C; Table S4), including the ERF, MYB, WRKY, bHLH, GRAS, NAC, etc. The ERF, WRKY, bHLH, and MYB families comprised the largest groups, with 41, 21, 18, and 13 members, respectively, under salt stress conditions. Among these genes, 18 out of 41 ERFs, 18 out of 21 WRKYs, 10 out of 18 bHLHs, and 8 out of 13 MYB TFs were upregulated, whereas the remainder were downregulated. Furthermore, members of the GRAS and NAC families were upregulated under salt stress (Figure 2C).

The KEGG enrichment analysis indicated DEmRNAs significant enrichment in several pathways, including phenylpropanoid biosynthesis (ko00940), starch and sucrose metabolism (ko04075), and the MAPK signaling pathway in plants (ko04016) within the roots (Figure 2D). Additionally, GO annotation analysis revealed that the primary biological processes (BP) associated with the response of sweetpotato roots to salt stress included the defense response and protein phosphorylation processes (Figure 2E). With respect to CCs, DEmRNAs were predominantly associated with the plasma membrane, extracellular region, and plasmodesmata. In terms of molecular function (MF), DEmRNAs were associated primarily with protein serine/triad kinase activity, peroxidase activity, and oxidoreductase activity.

### 2.3. Global Responses of miRNAs to Salt Stress

**Figure 3:**
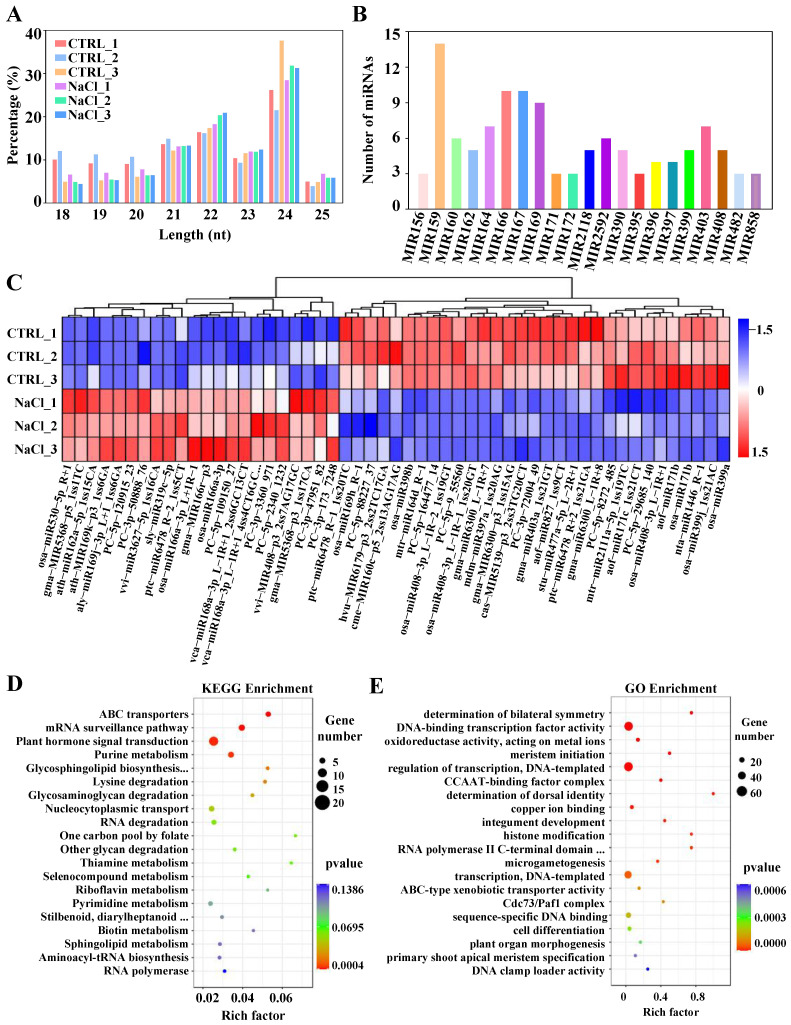
Identification and characterization of salt-responsive miRNAs in sweetpotato roots. (**A**) Length distribution of the identified miRNAs. (**B**) The distribution of known miRNAs in the miRNA family. (**C**) The expression patterns of differentially expressed miRNAs in sweetpotato roots. (**D**) KEGG enrichment results for target genes of differentially expressed miRNAs. (**E**) GO enrichment results for target genes of differentially expressed miRNAs in sweetpotato roots. The complete names for the omitted parts (…) are as follows: Glycosphingolipid biosynthesis—ganglio series; Stilbenoid, diarylheptanoid and gingerol biosynthesis; RNA polymerase II C-terminal domain phosphoserine binding.

To identify miRNAs responsive to salt stress in sweetpotato roots, six small RNA libraries were constructed and sequenced. A total of 49,990,009 and 35,346,360 raw reads were generated from the control and NaCl-treated root small RNA libraries, respectively (Table S5). After low-quality reads and joint sequences were filtered out, 31,470,023 clean reads were retained (Table S5). This study identified 458 miRNAs, including 267 known miRNAs and 191 potential novel miRNAs (Table S6). The identified miRNAs typically ranged from 18 to 25 nts in length (Figure 3A). The known miRNAs were categorized into 39 families, among which 21 families contain at least 3 members (Figure 3B), and miR159, miR166, and miR167 being the largest families, each containing more than ten members. Furthermore, 53 DEmiRNAs were identified between the control and NaCl-treated roots. Compared with those in the control roots, 22 miRNAs were upregulated, and 31 miRNAs were downregulated in response to salt stress (Figure 3C). The heatmap shows that vvi-MIR408-p3_2ss7AG17GC, vvi-miR3627-5p_1ss16CA, aly miR169j-3p_L+1_1ss6GA, ath-miR162a-5p_1ss15CA, and vca-miR168a-3p_L-1R+1_2ss6GC13CT presented the highest expression levels under salt stress, whereas osa-miR408-3p_L-1R+1, osa-miR169h_R-1, osa-miR399a, cas-MIR5139-p3_2ss3TG20CT, and PC-5p-88227_37 presented the lowest expression levels under similar conditions (Figure 3C).

The 857 putative target mRNAs were subsequently predicted via the psRNATarget server for the DEmiRNAs (Table S7). To further investigate the functions of the salt-responsive DEmiRNAs, KEGG enrichment and GO enrichment analyses were conducted on the target genes of the DEmiRNAs. KEGG enrichment analysis revealed that the target genes of the DEmiRNAs were mostly enriched in the plant hormone signal transduction pathway, followed by the mRNA surveillance pathway, ABC transporter pathway, and purine metabolism pathway (Figure 3D). The GO enrichment analysis indicated that the target genes were primarily involved in processes such as DNA-binding TF activity, and regulation of transcription (Figure 3E).

### 2.4. Global Responses of circRNAs to Salt Stress

**Figure 4:**
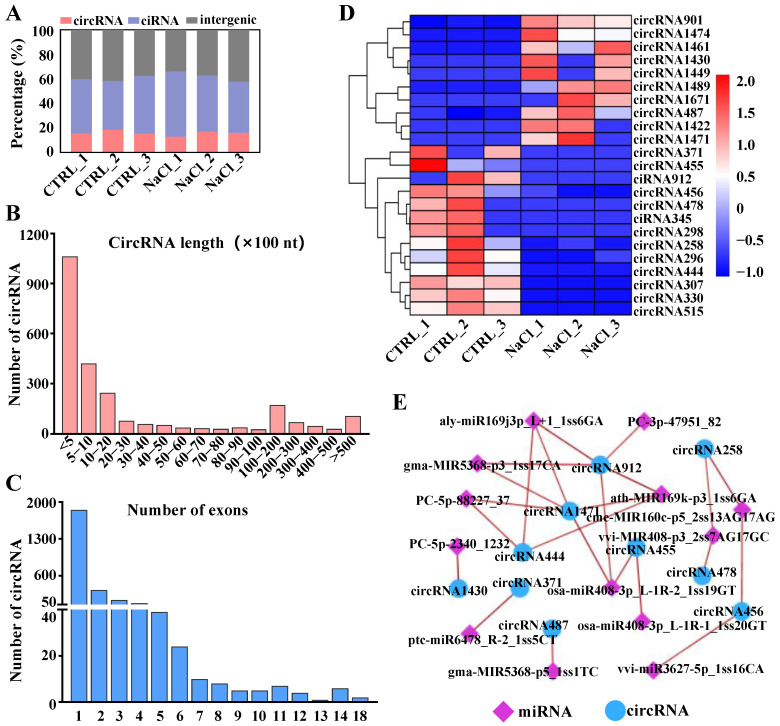
Identification and characterization of salt-responsive circRNAs in sweetpotato roots. (**A**) Type distribution of circRNAs. (**B**) Length distribution of circRNAs. (**C**) The number of exons in all identified circRNAs. (**D**) The expression patterns of the differentially expressed circRNAs. (**E**) The interaction network between differentially expressed circRNAs and differentially expressed miRNAs in sweetpotato roots.

A total of 2507 circRNAs were identified in the roots of sweetpotato (Table S8). The types of circRNAs present in different samples are illustrated in Figure 4A, revealing that most circRNAs were of the ciRNA type. Figure 4B shows the distribution of circRNA sequence lengths, with the majority measuring shorter than 2000 nts and approximately 42% being less than 500 nts. The number of exons in the circRNAs ranged from 1 to 18, with 1851 circRNAs (74%) containing only one exon, whereas only 3% of the circRNAs had more than six exons (Figure 4C). Additionally, 23 DEcircRNAs were detected, with 10 exhibiting upregulation and 13 exhibiting downregulation in sweetpotato roots under salt stress (Figure 4D). Among these, circRNA901, circRNA487, circRNA1422, circRNA1430, and circRNA1461 presented the highest expression levels under salt stress, whereas circRNA296, circRNA444, circRNA307, circRNA330, and circRNA515 presented the lowest expression levels under the same conditions (Figure 4D).

Studies have shown that circRNAs act as endogenous ceRNAs that influence the post-transcriptional regulatory functions of miRNAs. To analyze the interactions between circRNAs and miRNAs in greater depth, an interaction network of DEcircRNAs and DEmiRNAs was constructed. The results indicated that circRNA1471, circRNA912, and circRNA444 had more connections (≥3) than the other circRNAs did, suggesting their potential significance as ceRNAs within the network (Figure 4E).

### 2.5. Global Response of lncRNAs to Salt Stress

**Figure 5:**
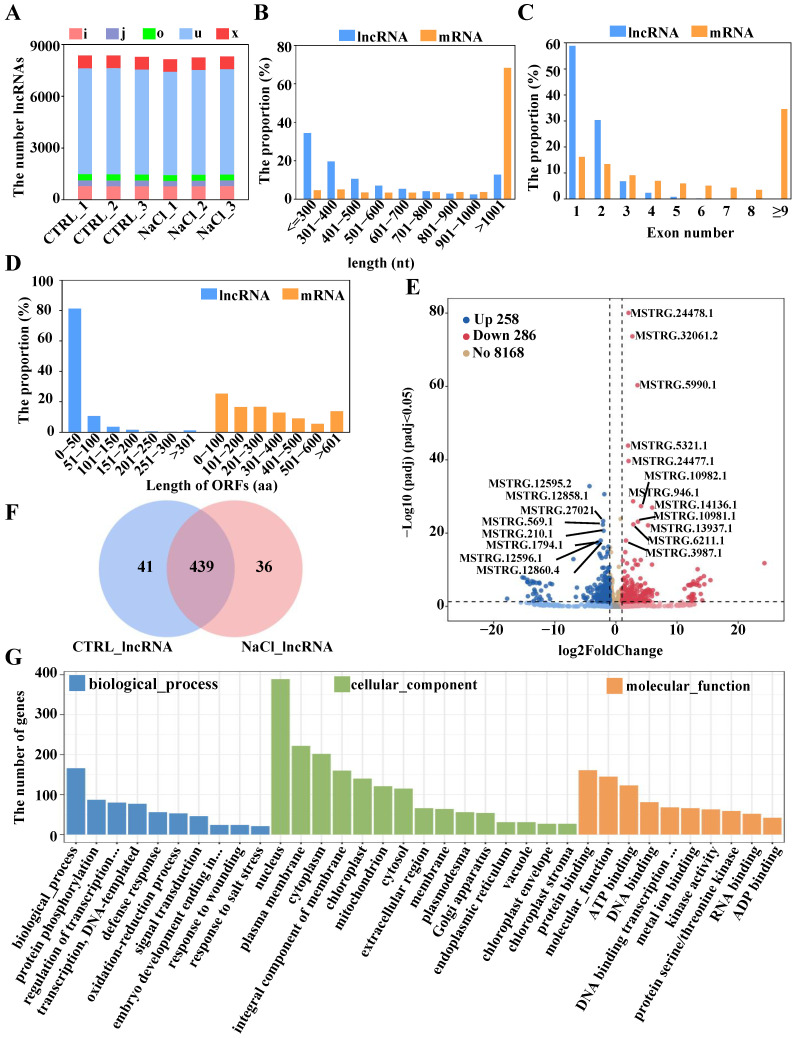
Identification and characterization of salt-responsive lncRNAs in sweetpotato roots. (**A**) Classification of lncRNAs in each sample. (**B**) Transcript length, (**C**) exon number, and (**D**) ORF length in lncRNAs versus mRNAs. (**E**) Volcano plot of the detected differentially expressed lncRNAs and nonsignificantly differentially expressed lncRNAs. (**F**) Venn diagram showing the number of differentially expressed lncRNAs in CTRL/NaCl. (**G**) GO annotation for targets of DElncRNAs in sweetpotato roots. The complete names for the omitted parts (…) are as follows: regulation of transcription, DNA-templated; embryo development ending in seed dormancy; DNA binding transcription factor activity.

To identify salt-responsive lncRNAs accurately, we obtained a total of 8712 novel lncRNAs via the Coding–Noncoding Index (CNCI, version 2.0) and Coding Potential Calculator (CPC2, version 0.9) software (Table S9). On the basis of the genomic position of these lncRNAs relative to protein-coding genes, we categorized them into five categories: ‘i’ for intronic lncRNAs, ‘j’ for bidirectional lncRNAs, ‘o’ for sense lncRNAs, ‘u’ for intergenic lncRNAs, and ‘x’ for antisense lncRNAs. As illustrated in Figure 5A, u-type lncRNAs were the most prevalent across all the samples. Prior studies have revealed distinct structural characteristics—such as sequence length, number of exons, length of the open reading frame (ORF), and expression levels—between lncRNAs and mRNAs. Therefore, this study compared the structural features and expression levels of lncRNAs and mRNAs.

Our findings revealed that the majority of lncRNAs were shorter than 600 nts, with 35% being shorter than 300 nts, whereas 68% of the mRNAs exceeded 1000 nts in length (Figure 5B). In terms of the number of exons, 89.29% of the lncRNAs contained one to two exons, with only 0.46% having more than six exons. In contrast, nearly all the mRNAs possessed exons, and 34.62% of the mRNAs contained at least nine exons (Figure 5C). Furthermore, the ORF length of lncRNAs is considerably shorter than that of mRNAs (Figure 5D; Tables S2 and S9).

We subsequently observed significant differences in lncRNA expression between the control and NaCl-treated roots. The volcano plot revealed 544 DElncRNAs in sweetpotato roots (Figure 5E). Among these DElncRNAs, 258 were upregulated, whereas 286 were downregulated in response to salt stress. Figure 5E highlights that the expression levels of the lncRNAs MSTRG.24478.1/32061.2/5990.1, etc., were significantly upregulated, whereas the expression levels of the lncRNAs MSTRG.12595.2/12858.1/27021.1, etc., were markedly downregulated under salt stress. Additionally, 41 lncRNAs were expressed specifically in the control roots, whereas 36 were unique to the NaCl-treated roots (Figure 5F).

LncRNAs are predicted to regulate the expression of nearby genes, leading to the identification of target genes for the cis-regulation of DElncRNAs. A total of 297 interaction pairs between DElncRNAs and DEmRNAs were identified (Table S10). Most of the DEmRNAs and DElncRNAs presented one-to-many matches, whereas a subset presented one-to-one matches (Table S10). To further investigate the potential functions of these DElncRNAs, GO annotation analysis was conducted on the DElncRNA-targeted genes (Figure 5G). The GO annotation of the DElncRNA targets in the roots revealed that 10 biological process terms were enriched (such as the protein phosphorylation (GO:0006468), regulation of transcription, DNA-templated (GO:0006355), transcription, DNA-templated (GO:0006351), etc.). Additionally, 15 cellular component terms were identified (mainly related to the nucleus (GO:0005634), plasma membrane (GO:0005886), an integral component of membrane (GO:0016021), etc.), along with 10 molecular function terms (mainly protein binding (GO:0005515), ATP binding (GO:0005524), DNA binding (GO:0003677), etc.) (Figure 5G).

### 2.6. CeRNA Regulatory Network in Response to Salt Stress

**Figure 6:**
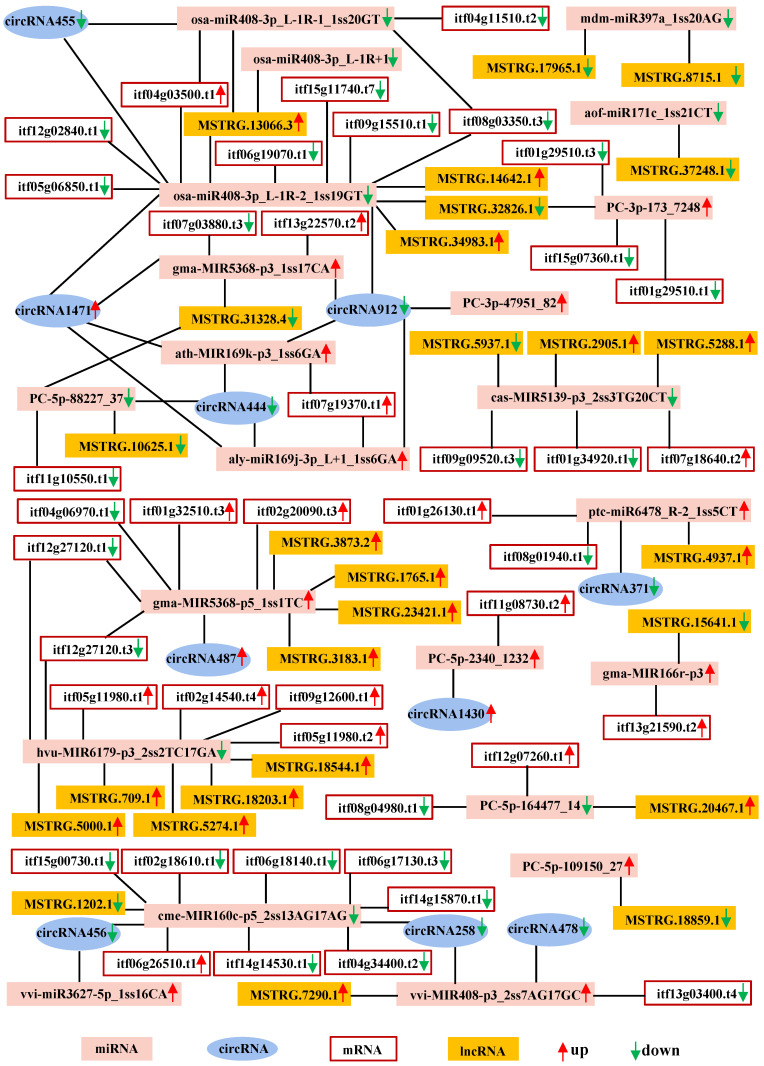
CeRNA network constructed with all salt-responsive differentially expressed mRNAs, lncRNAs, circRNAs, and miRNAs in sweetpotato roots.

To elucidate the global regulatory network of mRNAs and ncRNAs in sweetpotato subjected to salt stress, a ceRNA network was developed on the basis of the ceRNA hypothesis. This network consisted of 42 DEmRNAs, 22 DEmiRNAs, 27 DElncRNAs, and 10 DEcircRNAs. A total of 64 interaction pairs between DEmiRNAs and DEmRNAs, 32 DEmiRNA–DElncRNA pairs, and 27 DEmiRNA–DEcircRNA pairs were identified through screening (Figure 6; Table S11). Within the ceRNA network, osa-miR408-3p_L-1R-1_1ss20GT, osa-miR408-3p_L-1R-2_1ss19GT, gma-MIR5368-p5_1ss1TC, gma-MIR5368-p3_1ss17CA, hvu-MIR6179-p3_2ss2TC17GA, cme-MIR160c-p5_2ss13AG17AG, and cas-MIR5139-p3_2ss3TG20CT were involved in more than six nodes, suggesting that these miRNAs may play critical roles in the response of sweetpotato to salt stress. Furthermore, circRNA1471, circRNA912, circRNA444, lncRNA MSTR.13066.3, lncRNA MSTRG.31328.4, and lncRNA MSTRG.32826.1 bound to at least two miRNAs, indicating that they may function as miRNA sponges.

### 2.7. Analysis of Key Pathways of Sweetpotato Response to Salt Stress

**Figure 7:**
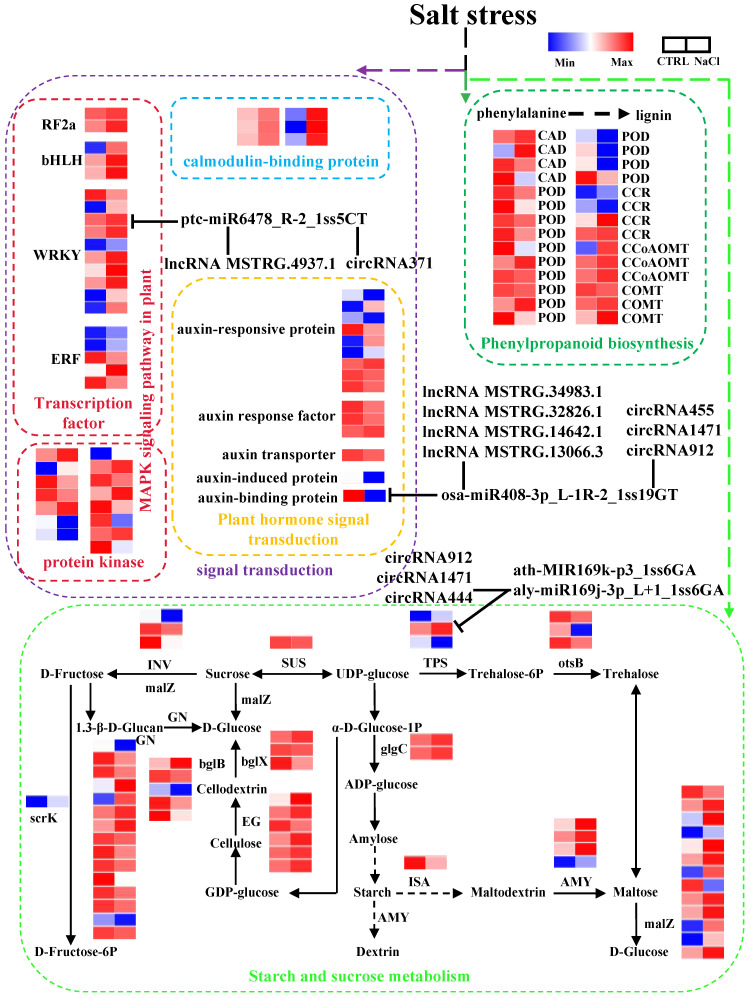
The important pathways of sweetpotato response to salt stress and the relationship between DEmRNAs and ceRNAs. Blue and red boxes represent lower and higher expression lever, respectively. CCoAOMT, caffeoyl-CoA 3-O-methyltransferase; COMT, caffeic acid 3-O-methyltransferase; CCR, cinnamoyl-CoA reductase; CAD, cinnamyl alcohol dehydrogenase; POD, peroxidase; INV, invertase; GN, glucan endo-1,3-β-glucosidase; SUS, sucrose synthase; bglX, β-glucosidase; bglB, β-glucosidase; malZ, α-glucosidase; EG, endoglucanase; AMY, α-amylase; TPS, trehalose 6-phosphate synthase; glgC, glucose-1-phosphate adenylyltransferase; otsB, trehalose 6-phosphate phosphatase; scrK, fructokinase. The T-shaped arrow indicates the predicted target gene of miRNA, and the solid line indicates that lncRNAs and circRNAs are predicted as ceRNA, competing with miRNA for the binding site. The solid arrow indicates that two substances are linked by one step reaction, and the dashed arrow indicates that there are more than one reaction bewteen the two substances.

According to the results of whole-transcriptome RNA sequencing, phenylpropanoid biosynthesis, starch and sucrose metabolism, MAPK signaling pathway in plants, and plant hormone signal transduction pathways are important in response to salt stress in sweetpotato (Figure 2D and Figure 3D). We found that 12 DEmRNAs related to phenylpropanoid biosynthesis were up-regulated, while 16 DEmRNAs were down-regulated (Figure 7; Table S12). In particular, 60 DEmRNAs were identified in the starch and sucrose metabolism pathway, among them, predicted *α-amylase* (*AMY)*, *glucose-1-phosphate adenylyltransferase* (*glgC)*, and *fructokinase* (*scrK)* genes were all up-regulated under salt stress, while *sucrose synthase* (*SUS)*, *invertase* (*INV*) were all down-regulated (Figure 7; Table S12). *Alpha, alpha-trehalose-phosphate synthase* (*TPS*) is the target gene of ath-MIR169k-p3_1ss6GA and aly-miR169j-3p_L+1_1ss6GA, and its expression is regulated by these two miRNAs together with circRNA912, circRNA1471 and circRNA444. In the plant hormones pathway, one auxin transport, one auxin-binding protein, and one auxin-induced protein were down-regulated, and nine auxin-responsive proteins have different expression patterns under salt stress. *Itf05g06850.t1* (predicted *auxin-binding protein ABP19a-like*) was predicted as the target gene of the key node osa-miR408-3p_L-1R-2_1ss19GT. Four lncRNAs (lncRNA MSTRG.34983.1, lncRNA MSTRG.32826.1, lncRNA MSTRG.14642.1, and MSTRG.13066.3) and three circRNAs (circRNA455, circRNA1471 and circRNA912) compete with osa-miR408-3p_L-1R-2_1ss19GT for binding the target gene. In addition, 20 TFs and 15 protein kinases were enriched in the MAPK signaling pathway plants, among which only 3 TFs were down-regulated under salt stress, while the other transcription factors were up-regulated (Figure 7). *Itf07g21360.t1* (probable WRKY transcription factor 57) was predicted as the target gene of ptc-miR6478_R-2_1ss5CT. And lncRNA MSTRG.4937.1 and circRNA371 were predicted as ceRNAs for ptc-miR6478_R-2_1ss5CT. Ca^2+^ signal transduction was essential for salt stress response in plants. In the study, we found that six DEmRNAs related to calmodulin (CaM) were all up-regulated under salt stress treatment. The complex regulatory mechanisms for the interaction between mRNAs and ncRNAs need to be further investigated.

## 3. Discussion

Salt stress significantly affects the growth and development of plants. To date, numerous studies have explored the functions and regulatory mechanisms of genes involved in salt stress. Although many protein-coding genes related to salt stress have been functionally characterized, the roles of noncoding genes, which may have significant regulatory implications, in sweetpotato subjected to salt stress remain largely unknown. In this study, we identified salt-responsive DEmRNAs, DEmiRNAs, DElncRNAs, and DEcircRNAs in sweetpotato roots under salt stress and established a salt-responsive ceRNA network on the basis of potential interactions among various RNAs.

### 3.1. Analysis of Salt-Responsive mRNAs in Sweetpotato

The response of plants to salt stress is a highly complex process that includes signal transduction, substance, and energy metabolism [81,82]. On the basis of the results of whole-genome sequencing, we initially examined the characteristics of mRNAs in sweetpotato roots in response to salt stress. A total of 814 mRNAs were found to be specifically expressed under salt stress conditions but not under normal growth conditions, suggesting their involvement in the salt stress response. Some genes associated with salt stress were upregulated, whereas others were downregulated. For example, the expression of the homologous gene *TRX* (*thioredoxin*) was upregulated under salt stress, whereas the expression of the homologous gene *MT2A* (*metallothionein*) was downregulated in sweetpotato. In *Arabidopsis*, overexpression of the *MT2A* gene enhances stress resistance by preserving chlorophyll, increasing the K^+^/Na^+^ ratio and proline content, and reducing the levels of reactive oxygen species (ROS) during salt stress [83]. The sweetpotato genes *TRX* and *KcTrxf* presented different expression patterns under salt stress, potentially indicating the activation of different pathways in response to this stressor.

Numerous TFs, including those in the bZIP, AP2/ERF, MYB, NAC, and WRKY families, play crucial roles in stress responses [84]. Yang et al. (2009) reported that *AtbZIP24* was induced by salt stress in *Arabidopsis* but was suppressed in the salt-tolerant relative *Lobularia maritima* [85]. Liu et al. (2007) noted that salt stress triggers a signaling cascade involving the processing of *AtbZIP17* in *Arabidopsis* [86]. In the present study, five bZIP TFs were induced under salt stress, suggesting that these bZIP TFs may play significant roles in sweetpotato. In *Arabidopsis*, high salinity markedly increased the expression of *ERF1 (ethylene-responsive transcription factor 1)*, with plants overexpressing *ERF1* demonstrating greater tolerance to salt stress [87]. However, 15 ERFs were downregulated in response to salt treatment in sweetpotato (Figure 2D; Table S4), indicating that these ERF TFs may not function as positive regulators in sweetpotato as they do in *Arabidopsis*. The overexpression of *WAKY25* and *WAKY33* has been shown to increase salt tolerance in *Arabidopsis*. In this study, 21 differentially expressed WRKY TFs were identified (Figure 2D; Table S4). The expression of the sweetpotato *WRKY* genes *WRKY7/22/26/28/31/45/48/53/57/61/71/75* was upregulated, whereas that of *WRKY9/31/70* was downregulated, suggesting that these genes may play different roles in the salt stress response in sweetpotato. Additionally, members of the MYB and bHLH families respond to ABA and ROS signals related to salt adaptation [84,88]. Several differentially expressed MYB and bHLH TFs were also identified in our study (Figure 2D; Table S4). Collectively, these results indicate that the differentially expressed TFs may be involved in the complex regulatory system governing the salt response in sweetpotato.

The functional analysis of DEmRNAs in the roots revealed that starch and sucrose metabolism was one of the predominant metabolic pathways under salt stress (Figure 2D). Sucrose metabolism is associated with increased sensitivity of plant development to abiotic stress, as a reduction in hexose can trigger downstream stress responses [89]. Moreover, stress can influence the expression of acidic β-fructofuranosidase, which plays a role in the metabolic signal transduction of primary metabolism and defense responses [90,91]. A number of mRNAs related to glycolysis, including predicted sucrose synthase (*itf02g07130*), alpha-trehalose-phosphate synthases (*itf11g03710* and *itf07g19370*), beta-fructofuranosidases (*itf13g04850* and *itf00g61200*), pyruvate kinase (*itf12g12910*), and beta-glucosidases (*itf08g18320*, *itf01g03560*, *itf01g03570*, and *itf01g26150*), were downregulated under salt stress in sweetpotato. Conversely, the expression of predicted phosphofructokinases (*itf03g18300* and *itf03g18310*) and glucoendo-1,3-beta-glucosidases (*itf04g09810*, *itf04g12060*, *itf04g12110*, and *itf04g12090*) was significantly upregulated. These results suggest that salt stress may hinder the gluconeogenesis pathway, whereas acetyl coenzyme A and oxaloacetic acid might be supplied to the tricarboxylic acid cycle through alternative routes. Collectively, these findings indicate that salt stress enhances the energy metabolism of sweetpotato root cells.

The leucine-rich repeat protein kinase (LRR) is crucial for the activation of signal perception and defense responses. Research on tobacco [92] and *M. truncatula* [93] has shown that LRR expression is upregulated in response to salt stress. In the present study, the expression of predicted LRRs (*itf10g19220*, *itf10g02800*, *itf10g02580*, *itf10g02700*, *itf07g07300*, *itf10g18790*, *itf01g03550*, *itf01g06180*, and *itf15g19970*) was also found to be upregulated under salt stress, suggesting that these LRRs are involved in signal transduction and defense responses in sweetpotato during salt stress.

### 3.2. Analysis of Salt-Responsive miRNAs in Sweetpotato

In plants, miRNAs play crucial roles in regulatory networks at both the transcriptional and post-transcriptional levels [14]. Numerous miRNAs have been identified as key players in plant responses to abiotic stress [94,95,96]. This study revealed a total of 458 miRNAs expressed in sweetpotato roots in response to salt stress, comprising 267 known miRNAs and 191 putative novel miRNAs (Table S6). The known miRNAs can be categorized into 39 miRNA families. Notably, certain members within the same family presented varying expression patterns under salt stress. For example, osa-miR408-3P_L-1R-1_1ss20GT, osa-miR408-3P_L-1R-2_1ss19GT, and osa-miR408-3P_L-1R+1 from the miR408 family were downregulated under salt stress, whereas the expression of vvi-MIR408-p3_2ss7AG17GC was upregulated. Similarly, the expression of aly miR169J-3P_L+1_1ss6GA and ath-miR169K-p3_1ss6GA increased in response to salt stress, whereas the expression of osa-miR169h_R-1 was downregulated. These findings suggest that miRNA members within the same family may have distinct functions in response to salt stress.

Although many miRNAs related to salt stress are conserved across various plant species, certain miRNAs exhibit different regulatory patterns among different species [97,98]. In both rice and *Arabidopsis*, increased salt stress can trigger the expression of members of the miR169 family, which play crucial roles in the response to salt stress [27]. However, the expression of sly-miR169e-3p is inhibited under salt stress and is significantly downregulated in both tomato varieties [14]. Similarly, in this study, the expression of aly-miR169J-3P_L+1_1ss6GA and ath-miR169K-p3_1ss6GA increased in response to salt stress, whereas the expression of osa-miR169h_R-1 was downregulated. These findings suggest that members of the miR169 family respond differently to salt stress across different species (Figure 3C).

In *Helianthus tuberosus*, treatment with 100 mM NaCl resulted in the upregulation of miR390 expression, whereas treatment with 300 mM NaCl led to its downregulation. Additionally, miR390 can be induced in poplar (*Populus* spp.) under salt stress [99,100]. Our results revealed that five members of the miR390 family presented different expression patterns under 200 mM NaCl treatment. Among them, osa-miR390-5p, osa-miR390-5p_1ss19GA, and gma-miR390b-5p were upregulated, whereas gma-miR390a-3p_R+1 and bdi-miR390a-3p_2ss17CT19CT were downregulated (Figure 3C). Furthermore, it has been reported that the miR397 family is inhibited by salt stress in *Carthamus tinctorius* and *S. lycopersicum* [101], which is consistent with the findings of our sweetpotato study.

The expression of miR408 is induced in *Arabidopsis* [102] and cotton [103], but it is significantly suppressed in rice [104], *M. truncatula* [105], and radish [106] under salt stress. In this study, the expression of osa-miR408-3p_L-1R+1, osa-miR408-3p_L-1R-2_1ss19GT, osa-miR408-3p_L-1R-1_1ss20GT, and zma-miR408b-5p_L+1R-1_1ss3AG was downregulated under salt stress treatment (Figure 3C). These findings indicate that the salt response mechanism of miR408 in sweetpotato may be similar to that in *Oryza sativa*, *M. truncatula*, and *Raphanus sativus*. In *Arabidopsis* and maize, the expression of miRNA168 is significantly induced under salt stress [26,107]. Our results also revealed that the expression of miRNA168a/b was upregulated under salt stress (Table S6). Therefore, it is essential to analyze the miRNAs of specific species in relation to salt stress.

To increase our understanding of the regulatory role of sweetpotato miRNAs in response to salt stress, we analyzed the identified miRNAs and their target genes. A total of 857 target genes of 47 DEmiRNAs were predicted (Table S7). These target genes include TFs, hormone response genes, DNA/RNA binding proteins, protein-coding genes, and enzymes, indicating that these mRNAs may be influenced by miRNAs in the context of salt stress.

The NAC family is the most extensive TF family in plants and is crucial for responses to abiotic stress [108]. The OsNAC5 protein binds to the promoter region of OsLEA3, leading to the upregulation of stress-related gene expression, thereby increasing stress tolerance in rice [109]. Additionally, GmNAC021 is significantly expressed in a drought-resistant soybean variety, suggesting its involvement in soybean leaf development and the plant’s response to drought stress [110]. Our findings indicate that mtr-miR164d_R-1 is significantly downregulated under salt stress and is predicted to target NAC7. This downregulation of mtr-miR164d_R-1 may promote the expression of NAC7, thereby contributing to the regulation of the sweetpotato response to salt stress.

Moreover, the increased expression of the target gene *HD-ZIP* and decreased levels of miR166 in salt-tolerant soybeans could increase salt tolerance [111,112]. A study has also shown that stu-miR166b, which is significantly downregulated due to salt stress, targets *ATHB-14/15* [79]. Our research revealed that the expression of osa-miR166a-3p_L+1R-1 and osa-miR166a-3p was upregulated under salt stress, whereas the expression of the predicted target gene, homeobox-leucine zipper protein *ATHB-15-like*, did not significantly change under salt stress conditions. This suggests considerable variability in the salt response of miRNAs among different species. The target TFs of miR171 have also been shown to play a role in the regulation of gene expression and signal transduction, which may contribute to stress responses [31,113]. In this study, miR171b/c were predicted to target GRAS TFs. A substantial body of research has indicated that GRAS TFs are vital for plant adaptation to adverse environmental conditions [114,115,116]. Therefore, miR171 may play a crucial role in the response of sweetpotato to salt stress.

### 3.3. Analysis of Salt-Responsive circRNAs in Sweetpotato

As a newly characterized class of ncRNAs, circRNAs have been identified in numerous plant species, including *Arabidopsis* [117,118], rice [119,120], tomato [121,122], wheat [59], soybean [123], potato [124], tea [125], and cucumber [68,126], and they play significant roles in plant stress responses. Zhu et al. (2019) identified 1934 circRNAs in cucumber roots and 44 in leaves, with differential regulation under salt stress [126]. However, the impact of salt stress on the expression of circRNAs in sweetpotato remains poorly understood. This study identified 2507 circRNAs from sweetpotato roots. Notably, 23 circRNAs presented different expression patterns between control roots and NaCl-treated roots, indicating their significant role in mediating the response to salt stress (Table S8). Recently, certain circRNAs have been shown to possess miRNA binding sites, which may allow them to act as sponges that sequester miRNAs, thereby preventing these miRNAs from interacting with their target mRNAs [124,127]. This study predicted that 10 DEcircRNAs may function as miRNA sponges in response to salt stress in sweetpotato roots. Among these DEcircRNAs, circRNA444, circRNA1471, circRNA912, circRNA258, and circRNA455 interact with multiple miRNAs, suggesting that these DEcircRNAs may serve as important regulators of salt stress responses in sweetpotato.

### 3.4. Analysis of Salt-Responsive lncRNAs in Sweetpotato

Increasing evidence supports the significant role of lncRNAs in the stress response [49,128,129,130]. However, only a limited number of lncRNAs are involved in the response to salt stress. To further elucidate the function of lncRNAs in sweetpotato under salt stress, we identified a total of 8712 novel lncRNAs from sweetpotato roots subjected to both normal conditions and salt stress through whole-transcriptome sequencing (Table S9). Compared with protein-coding genes, lncRNAs are generally shorter in length and present fewer exons in their structure, which is consistent with prior studies [131,132]. Under salt stress, 258 lncRNAs were upregulated, whereas 286 were downregulated, suggesting that these lncRNAs may play crucial roles in the salinity response. As transcriptional regulators, lncRNAs can directly or indirectly influence the expression of functional genes [44,133]. This study identified 297 pairs of cis-lncRNA–mRNA interactions. Among these, 190 pairs presented a positive correlation at the expression level, whereas only 107 pairs presented a negative correlation (Table S10). To gain further insight into the functions of these DElncRNAs under salt stress, we conducted GO term enrichment analysis on their target transcripts. The results indicated that most target transcripts were significantly enriched in processes such as dioxygenase activity, defense response, oxidation–reduction processes, protein phosphorylation, signal transduction, response to wounding, and response to salt stress. These findings suggest that lncRNAs are actively involved in the salt response in sweetpotato.

### 3.5. CeRNA Networks May Provide New Light on the Regulatory Function of ncRNAs

The ceRNA hypothesis has gained substantial acceptance since its introduction a few years ago [63]. Although significant advancements have been made in understanding human diseases through the ceRNA hypothesis [134], research in the plant remains comparatively limited. Within the ceRNA network, miRNAs play crucial roles in linking and regulating various RNA molecules. This study predicted interactions involving 63 DEmiRNAs and DEmRNAs, 31 DEmiRNAs and DElncRNAs, and 62 DEmiRNAs and DEcircRNAs, resulting in the construction of a ceRNA network (Figure 6). The key nodes identified within this network included osa-miR408-3p_L-1R+1, osa-miR408-3p_L-1R-1_1ss20GT, osa-miR408-3p_L-1R-2_1ss19GT, aof-miR171c_1ss21CT, mdm-miR397a_1ss20AG, vvi-MIR408-p3_2ss7AG17GC, gma-MIR166r-p3, ath-MIR169k-p3_1ss6GA, and aly miR169j-3p_L+1_1ss6GA, indicating their potential importance in the salt response of sweetpotato.

Previous studies have primarily associated miR408 with stress responses [135,136]. In *Medicago truncatum*, miR408 was found to be significantly upregulated in response to drought stress [136]. In sweetpotato, increased expression of miR408 has been associated with reduced resistance to insect feeding [76]. Conversely, in chickpeas, the overexpression of miR408 increased drought tolerance [135]. In rice, miR408 plays a role in regulating grain yield and photosynthesis through phytocyanin [137]. Within the ceRNA network, core nodes, such as osa-miR408-3p_L-1R+1, osa-miR408-3p_L-1R-1_1ss20GT, osa-miR408-3p_L-1R-2_1ss19GT, and vvi-MIR408-p3_2ss7AG17GC, connect to 19 potential ceRNAs (Figure 6; Table S11), suggesting that miR408 may also contribute significantly to the response to salt stress.

Recently, the miR169-NF-YA module was shown to regulate the auxin-mediated response to cold stress in the roots of *Arabidopsis* [98,138]. These findings indicated that ath-MIR169k-p3_1ss6GA and ly miR169j-3p_L+1_1ss6GA were important core nodes in the ceRNA network, with their expression levels increasing under saline conditions, suggesting their potential roles in the salt stress response. Notably, both miRNAs targeted *itf07g19370.t1*, which encodes a predicted alpha-trehalose-phosphate synthase, a key enzyme involved in starch and sucrose metabolism (Figure 7). These findings suggest that miR169s may play a role in sugar metabolism in response to salt stress.

Cme-MIR160c-p5_2ss13AG17AG is another miRNA identified as a core node in the ceRNA network. It is predicted to target various genes, including *itf06g18140.t1* (predicted germin-like protein), *itf06g26510.t1* (predicted protein NRT1/PTR FAMILY 2.11-like), *itf15g00730.t1* (predicted GDSL esterase/lipase at5g03820-like isoform x2), and other proteins (*itf14g15870.t1*, *itf02g18610.t1*, and *itf06g17130.t3*). Research has shown that germin-like proteins are ubiquitous and play crucial roles in plant responses to various abiotic stresses [139]. The germin-like protein-encoding gene has been shown to have high expression levels in the rice cultivar Super Basmati under salt and drought stress [139]. NRT1/PTR FAMILY was originally characterized as a transporter of nitrate and peptides; recent studies have also revealed its role in transporting plant auxins, ABA, gibberellins, and secondary metabolites [140]. Additionally, GDSL-type esterases/lipases carry out essential functions in plants, particularly in response to biotic and abiotic stresses [141]. Collectively, these findings suggest that Cme-MIR160c-p5_2ss13AG17AG may target multiple mRNAs and engage in the response to salt stress through various pathways. Furthermore, the ceRNA network included several previously unreported miRNAs (hvu-MIR6179-p3_2ss2TC17GA, vvi-miR3627-5p_1ss16CA, gma-MIR5368-p5_1ss1TC, and gma-MIR5368-p3_1ss17CA) along with several novel miRNAs (PC-3p-173_7248, PC-3p-47951_82, PC-5p-88227_37, PC-5p-2340_123, PC-5p-109150_27, and PC-5p-164477_14), which may also be involved in the salt stress response through unknown mechanisms.

In plants, miRNAs serve as negative regulators, repressing transcript translation through base complementarity with target mRNAs [142,143,144]. Increasing evidence suggests that lncRNAs and circRNAs function as miRNA sponges, playing crucial roles in various biological processes [145,146,147]. This study identified 26 DElncRNAs and nine DEcircRNAs within the ceRNA network, indicating that these ncRNAs may also act as miRNA sponges (Figure 6; Table S11). For example, circRNA455, circRNA1471, circRNA912, lncRNA MSTRG.13066.3, lncRNA MSTRG.14642.1, lncRNA MSTRG.32826.1, and lncRNA MSTRG.34983.1 may function as miRNA sponges by binding to osa-miR408-3p_L-1R-2_1ss19GT (Figure 7), osa-miR408-3p_L-1R-1_1ss20GT, gma-MIR5368-p3_1ss17CA, and ath-MIR169k-p3_1ss6GA, thereby regulating the expression of *itf06g19070.t1* (predicted polygalacturonase), *itf12g02840.t1* (predicted LRR receptor-like serine/threonine-protein kinase), *itf05g06850.t1* (predicted auxin-binding protein ABP19a-like), *itf15g11740.t7* (predicted protein BONZAI 3), *itf09g15510.t1* (predicted aspartyl protease AED3-like), and *itf08g03350.t3* (predicted protein RCC2), *itf04g11510*. However, notably, the majority of the DElncRNAs were not included in the ceRNA network. This observation led us to propose that these lncRNAs may be involved in the salt stress response through alternative pathways, possibly by inducing DNA methylation, regulating chromatin modifications, or acting as transcriptional enhancers.

## 4. Materials and Methods

### 4.1. Plant Materials and Treatments

The sweetpotato (*Ipomoea batatas* L.) variety Taizhong 6 was jointly cultivated by the Institute of Plant Physiology and Ecology in the Shanghai Academy of Life Sciences of CAS and Tai’an Academy of Agricultural Science. Taizhong 6 is an edible sweetpotato cultivar that has a good taste due to its high sweetness and carotenoids, but low fiber contents. In this study, the plant material Taizhong 6 seedlings were obtained from the Xuzhou Institute of Agricultural Sciences in Jiangsu Xuhuai District, China. Six-leaf-stage sweetpotato seedlings exhibiting comparable growth were selected and placed in half-strength Hoagland solution. The seedlings were cultured for one week in a growth chamber under a 16/8 light photoperiod at 28 °C. On the 8th day, the seedlings were evenly selected and randomly divided into two groups: the control group and the NaCl-treated group. The control group was cultured in a half-strength Hoagland solution, whereas the NaCl-treated group was cultured in a half-strength Hoagland solution containing 200 mM NaCl for 3 days. At 0, 24, 48, and 72 h, the roots of the seedlings from both groups were collected. These materials were used for the determination of physiological indicators or quickly frozen in liquid nitrogen and stored at −80 °C for RNA extraction.

### 4.2. Measurement of Physiologic Indicators

The contents of MDA, as well as anti-O_2_^.−^ activity and LOX activity, were measured following previously established protocols [73]. The MDA concentration was expressed in nmol g^−1^ on a fresh weight basis. The activities of both the anti-O_2_^.−^ and LOX enzymes were reported as U g^−1^ on a fresh weight basis. Specifically, the change in the value of O_2_^.−^ inhibited by 1 g tissue at 37 °C for 40 min was equivalent to that inhibited by 1 mg vitamin C, which was designated as one unit (U). Furthermore, one unit (U) of LOX activity was defined as the amount of enzyme required to catalyze the absorbance change of 0.001 units per minute by 1 g of tissue at 25 °C in a 1 mL system. All the experiments were conducted with three independent biological replicates.

### 4.3. RNA Extraction, Library Preparation, and RNA Sequencing

After salt stress treatment for two days, the roots from 10 sweetpotato seedlings were collected and mixed as one biological repeat. Three independent biological repeats were prepared separately for the control (without NaCl treatment) and the NaCl-treated groups. Total RNA was extracted from the control and NaCl-treated roots via TRIzol reagent (Invitrogen, Carlsbad, CA, USA) according to the manufacturer’s protocol. The quality and integrity of the RNA were assessed. After the depletion of ribosomal RNA, six libraries are designed as CTRL_1, CTRL_2, and CTRL_3 in the control group, and NaCl_1, NaCl_2, and NaCl_3 in the salt stress treatment group. Upon satisfactory completion of quality inspections, the libraries were sequenced via the Illumina NovaSeq™ 6000 platform (Illumina, San Diego, CA, USA), with a reading length of 2 × 150 bp (PE150) at both ends. For small RNA sequencing, another six small RNA libraries were prepared via the TruSeq Small RNA Sample Prep Kit (Illumina, San Diego, CA, USA), and the resulting small RNA fragments were sequenced on the Illumina HiSeq 2000/2500 platform (Illumina, San Diego, CA, USA), yielding a read length of 1 × 50 bp. All sequencing experiments have three biological repeats.

### 4.4. Identification and Analysis of mRNAs

To ensure accurate and reliable analytical results, Cutadapt (version 1.10) was used to remove sequencing adapters and low-quality sequencing data. HISAT2 (version 2.0.4) was subsequently used to align the preprocessed valid data against the sweetpotato reference genome (http://sweetpotato.uga.edu/ (accessed on 10 January 2023)). Statistical analyses were conducted on the basis of the gene position information specified in the genome annotation file, with reads assembled via StringTie (version 2.1.6). Following the generation of the final transcriptome, the expression levels of all transcripts were estimated via the R packages DESeq2 (version 3.2.5) and edgeR. Transcript expression levels were determined via the fragments per million exons per thousand bases (FRKM) method. A cutoff threshold of |log2FC| ≥ 1 and *p*-value < 0.05 was established for DEmRNAs. GO annotation and functional enrichment analyses for DEmRNAs were conducted via an online database (https://geneontology.org/ (accessed on 21 January 2023)), whereas KEGG enrichment analysis was performed via the Omicstudio online platform (https://www.omicstudio.cn/tool (accessed on 3 August 2024)).

### 4.5. Identification and Analysis of lncRNAs

To identify novel lncRNAs, the transcript assembly software StringTie (version 2.1.6) was used to assemble the reads. Initially, transcripts that overlapped with known mRNAs and novel mRNAs possessing coding potential were removed. Transcripts shorter than 200 bp and those with read coverage of less than 3 bp were subsequently filtered out. The remaining transcripts were then assessed for coding potential via CPC (version 0.9) and CNCI (version 2.0). Transcripts with a CPC score of less than 0.5 and a CNCI score of less than 0 were eliminated, and the remaining transcripts were classified as lncRNAs. All recognized lncRNAs were categorized into intronic lncRNAs, bidirectional lncRNAs, sense lncRNAs, intergenic lncRNAs, and antisense lncRNAs via the Cuffcompare program within the Cufflinks suite. The expression levels of the lncRNAs were quantified primarily via FPKM. DELncRNAs were identified on the basis of absolute values of log2FC ≥ 1 and a false discovery rate (FDR) < 0.05 via EdgeR. To explore the function of lncRNAs, the cis target genes of the DElncRNAs were predicted. In this study, a Perl script was used to screen for genes located within 100 kb upstream and downstream of the lncRNAs as cis targets. All cis-target genes of the DElncRNAs were then subjected to BLAST against the GO database to calculate the number of genes associated with each term.

### 4.6. Identification and Analysis of circRNAs

On the basis of the structural features and splicing characteristics of circRNAs, CircExploiter 2 (version 2.2.6) and CIRI (version 2.0.2) were utilized for the identification of circRNAs, with results from both software packages integrated. CircRNAs were identified according to the following criteria: a maximum of 2 mismatches, at least one back-spliced junction read, and a distance of less than 100 kb between two splicing sites in the genome. The expression levels of the circRNAs were determined via the split reads per billion mappings (SRPBM) method. The R package EdgeR was employed to identify DEcircRNAs that met the criteria of log2|FoldChange| ≥ 1 and a *p*-value < 0.05. Heatmaps of the DEcircRNA expression profiles were generated via MeV (version 4.9.0). The interactions between DEcircRNAs and DEmRNAs were predicted via Ssearch36 (version 36.3.6), and a visual network was constructed via Cytoscape (https://cytoscape.org (accessed on 13 August 2024)) [148].

### 4.7. Identification and Analysis of miRNAs

The raw data obtained from sequencing were processed via ACGT101-miR (LC Sciences, Houston, TX, USA) to eliminate 3′ adapters and junk reads, retaining only those reads with a base length of 18–25 nts. NonmiRNA sequences (including rRNA, snoRNA, snRNA, tRNA, and other RNA) present in the cleaned data were discarded through a comparative analysis with the Rfam database. The remaining reads were then mapped to specific species precursors in miRBase 21.0 to identify both known and novel miRNAs. The expression levels of the miRNAs were quantified via normalized values on the basis of the raw read counts of the miRNAs. DEmiRNAs were extracted via DEGseq, with absolute values of log2FC ≥ 1 and *p* < 0.05. The target genes for the DEmiRNAs were predicted via GSTAr (version 1.0) [149], which employs RNAplex program to predict the complementary pairing associations between miRNAs and their target genes, whereas the minimum free energy (MFE) was calculated to determine the optimal base pairing associations (MFE ratio > 0.65). Furthermore, the GO terms and KEGG pathways for the miRNA targets were annotated by referencing the GO database and KEGG pathway database.

### 4.8. Construction and Analysis of ceRNAs Regulatory Network

To elucidate the interactions among DEmRNAs, DEcircRNAs, DEmiRNAs, and DElncRNAs, we predicted the targeted binding associations among miRNAs, mRNAs, lncRNAs, and circRNAs, thereby constructing a regulatory network based on the ceRNA hypothesis. RNAplex program was utilized to predict the complementary pairing relationship between miRNAs and mRNAs. The MFE was calculated according to the thermodynamic structure to predict the optimal base pairing associations, with a cutoff for the MEF ratio (MFEratio) > 0.65 and AllenScore value ≤ 7. Subsequently, Ssearch36 (version 36.3.6) software was utilized to predict the interaction between miRNAs and ncRNAs. The target mimics filtering meets the following rules: (1) The bulge must be on the ncRNA and in the middle of the miRNA; (2) Four mismatches are allowed at most except the middle position of the miRNA, and no more than two consecutive mismatches are allowed [66]. Finally, we employed Cytoscape software (version 2.8) to visualize the regulatory associations [148].

## 5. Conclusions

Coding and noncoding RNAs, along with their interactions, play crucial roles in how plants respond to salt stress. The identification of ncRNAs has profound significance for agriculture and breeding, especially in revealing the molecular regulatory mechanisms of agronomic traits, improving production performance, and optimizing stress resistance. Our study provides a comprehensive analysis of the characteristics of noncoding RNAs in sweetpotato subjected to salt stress, highlighting the importance of their interactions in regulating the salt stress response. Our findings suggest that the identified miRNAs, lncRNAs, and circRNAs in sweetpotato may influence various cellular processes, including cell wall and plasma membrane permeability, protein phosphorylation, TF activity, and several metabolic pathways. These results pave the way for further investigations into the potential functions and mechanisms of salt-responsive ncRNAs and provide new targets for sweetpotato breeding to generate salt stress-tolerant cultivars through the manipulation of the miRNAs, circRNAs, and lncRNAs in the future.

## Data Availability

The data presented in this study are available on request from the corresponding author.

## Supplementary Materials

The following supporting information can be downloaded at: https://www.mdpi.com/article/10.3390/ijms26041660/s1.
